# Acute Supplementation of Yerba Mate Extract Did Not Change Muscle Strength in Physically Active Men Following the Strength Muscle Test: A Pilot Clinical Trial

**DOI:** 10.3390/nu14132619

**Published:** 2022-06-24

**Authors:** Patrícia C. B. Lobo, Débora D. da Silva, Gustavo D. Pimentel

**Affiliations:** Faculty of Nutrition, Federal University of Goiás, Goiânia 74605-080, Brazil; patriciacristina.nutri@gmail.com (P.C.B.L.); deboraadds@discente.ufg.br (D.D.d.S.)

**Keywords:** dietary supplements, Ilex paraguariensis, muscle strength, resistance training

## Abstract

Polyphenol supplementation may be useful during exercise. However, there is no evidence indicating yerba mate (YM) increases muscle strength. Thus, this study sought to evaluate the effect of acute YM supplementation on muscle strength following the strength test. In a crossover and pilot clinical trial, ten men were divided into two groups, receiving either supplementation with YM or a placebo. One hour after consumption of beverages, the participants were submitted to tests of one-repetition maximum (1 RM) on the bench press and leg press. The average age of the participants was 25.5 ± 4.1 years, and the average body mass index was 24.4 ± 2.9 kg/m². YM was not able to increase muscle strength when compared to the placebo in either the 1RM leg press exercise (YM: 225 ± 56.2 kg, vs. placebo: 223 ± 64.3 kg, *p* = 0.743, Cohen’s d = 0.03) or in the 1 RM bench press exercise (YM: 59.5 ± 20.7 kg vs. placebo: 59.5 ± 21.5 kg, *p* = 1.000, Cohen’s d = 0.) In conclusion, acute intake of YM did not change muscle strength in physically active men.

## 1. Introduction 

In recent years, increased use of dietary supplements, such as nitrate, caffeine, creatine, protein and polyunsaturated fatty acids, has been observed, since they also contribute to the modulation of body composition and physical performance [1].

Thus, supplements based on natural herbs, such as yerba mate (YM), have been studied because they have antioxidant, anti-inflammatory, vasodilator, hypocholesterolemic and hypoglycemic effects [2]. YM (Ilex paraguariensis) has phenolic components, such as xanthines (caffeine and theobromine), which stimulate the central nervous system [3], and saponins (ursolic and oleanolic acids), which are studied for their antifungal, insecticidal, anti-hemintic, cytotoxic, anti-inflammatory, immunostimulating, hypocholesterolemic and hypoglycemic properties [4]. Evidence points out that biological activities involving mate tea affect the loss of body mass and appetite control [5,6]; however, the mechanisms of action are still unknown.

Herbal supplements are believed to attenuate exercise-induced oxidative stress in athletes as they stabilize oxidative damage through neutralization of free radicals [7]. Panza et al. [8] evaluated the effect of consuming 600 mL/day of mate tea on muscle strength recovery and oxidative stress markers in men undergoing eccentric exercise for 11 days. It was observed that mate tea accelerated the recovery of muscle strength over 24 h after eccentric exercises were performed and increased the concentration of antioxidant compounds in the bloodstream, which may benefit individuals who need to perform exercises that involve the same muscle group as was damaged.

Thus, although the use of mate tea in exercise is suggested due to its antioxidant and/or anti-inflammatory properties, the mechanisms of action, as well as the recommended dose, are still unclear [9]. In addition, there is no concrete information about possible side effects of using mate tea during exercise [7]. Thus, in view of the scarcity in the literature of studies evaluating the effects of YM on bodybuilders, the objective of this study was to evaluate the effect of acute supplementation of YM on muscle strength following the strength test. 

## 2. Methods 

### 2.1. Study Design, Recruitment and Ethical Procedures

This was a crossover pilot clinical study carried out in a private gym in Goiânia, Goias, Brazil. The research consisted of eight meetings, in the morning, which took place from September to October 2021. The participants were divided into two groups in order to better conduct and organize the protocols (Supplementary Materials Figure S1).

**Figure 1:**
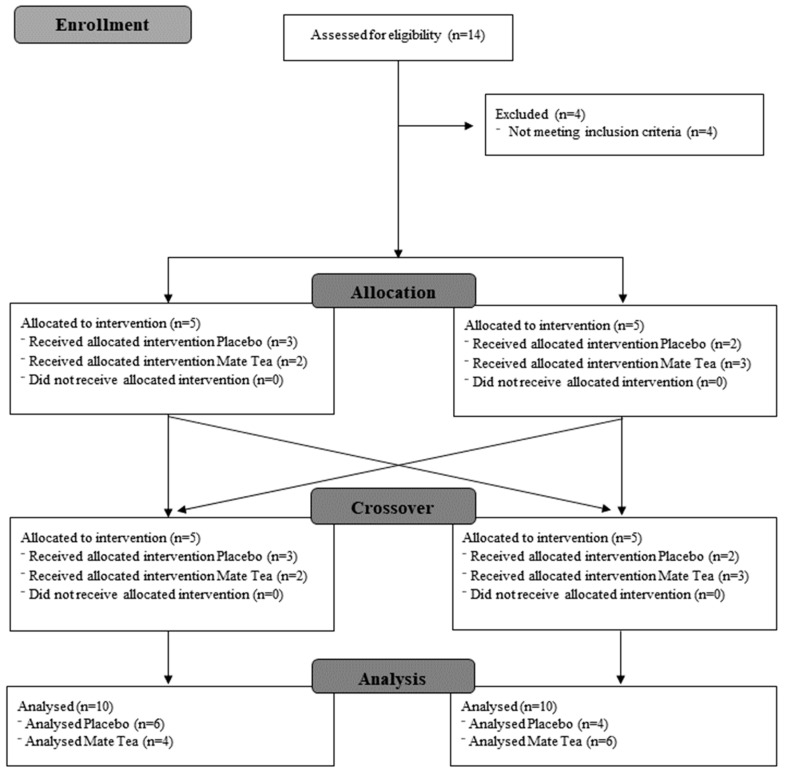
Study design.

Participants were recruited at the gym where the study was carried out, and also through social networks. A total of 14 individuals responded to the call, 10 of whom were included in the study and divided into one of two groups, receiving either YM or the placebo (Figure 1).

The design of this study was previously approved by the Local Research Ethics Committee (Number: 3,031,999). All participants were instructed about the study, and those who agreed to participate signed the Free and Informed Consent Term, in accordance with the National Health Council of the Ministry of Health of Brazil.

Only men were included in the study, all of whom had at least two months of experience with resistance exercise, were aged 18 years or over and were available to attend the gym on the days and times established for data collection. The exclusion criteria were smokers; having any disease; inflammatory and/or infectious conditions; injuries in the upper and/or lower limbs, which would have made it impossible to apply the strength tests; and allergy or intolerance to the supplement under study or to any of its isolated components, such as caffeine (Figure 1).

Based on the study by Panza et al. [8] (n = 12), considering a 20% sample loss (n = 2–3), a sample size (n = 10) for the crossover study was considered for this study. Fourteen men were recruited; however, there was a loss of 28.6% (n = 4). This method was adopted because there are no investigations in the literature regarding the effects of mate tea on muscle strength and in order to serve as a reference for calculating the sample size.

### 2.2. Experimental Procedures 

In the first two meetings, the loads of the individuals in the test of one-repetition maximum (1RM) on the bench press and on the leg press were estimated, and in the third and fourth meetings, the values found for the 1RM tests were confirmed. The fifth and sixth meetings involved the first application of supplementation or placebo, in which half of the individuals ingested YM and the other half the placebo. In the seventh and eighth meetings, the second supplementation or placebo was given, inverting the drink ingested and characterizing the crossover (Supplementary Materials Figure S1). The estimation of the 1RM, its confirmation and the application of the tests were carried out and supervised by a Physical Education professional.

In the last four meetings, referring to the application of the supplementation or placebo, the individuals were instructed to suspend the consumption of foods and products that are sources of caffeine in the 24 h prior to the test days, in addition to not performing vigorous activities in this period. Individuals were also instructed to go to the gym after 12 h of fasting. At the test site, participants received a standardized snack consisting of a flour source, a fat source and a dairy drink, totaling 145 g (bread 50 g—margarine 5 g—coconut-flavored yogurt 90 g), and providing 267.5 kcal, 7.5 g of protein, 40.9 g of carbohydrates and 7.8 g of lipids.

At the gym, before eating the standardized snack, an anthropometric assessment was performed to obtain the weight and height of the participants. Then, after the consumption of the snack, the mate tea or placebo was offered, and an interval of one hour after consumption of the drink was waited for the participants to perform the strength tests. During this interval, the 24 h food recall and the caffeine intake assessment questionnaire were applied, and the participants were also asked about which beverage they believed they had ingested, the mate tea or placebo.

### 2.3. Supplementation

Soluble mate tea and a powdered industrialized soft drink (placebo) were weighed using a Shimadzu^®^ brand analytical balance and stored in plastic bags.

In the fifth and sixth meetings (Supplementary Materials Figure S1), referring to the beginning of supplementation, half of the participants received opaque black disposable bottles, with a lid and straw, containing 3 g of soluble YM (Matte Leão^®^, Valinhos, Brazil), diluted in 250 mL of cold mineral water. The other half of the participants received the placebo drink, containing 1.6 g of powdered flavored tea with lemon (Ajinomoto^®^, Limeira, Brazil), without sugar, diluted in 250 mL of cold mineral water. The flasks were dark in color so the difference between drinks would not be noticeable. Between the first and second application of supplementation, a washout period of 14 days was respected.

Participants and researchers were not aware of which groups were ingesting each compound. Each cup containing the supplementation or placebo was identified with a sticker (A or B) prior to random delivery of cups to participants. Only at the end of the study was the content of the cups revealed to the researchers.

### 2.4. Muscle Strength Test

To assess muscle strength, one-repetition maximum (1RM) was performed using bench press and leg press exercises.

The tests were performed in duplicate (test and retest) in the first four meetings, with an interval of seven days (Supplementary Materials Figure S1). To reach the 1RM load, the subjects were instructed to perform a warm-up of 10 repetitions with no assigned load with a three-minute rest, followed by another 10 repetitions at 50% of the predicted RM load. Soon after, the loads were adjusted to the devices and the individuals were instructed to perform one repetition, with the load being increased later and the procedure repeated up to five times, with a five-minute rest interval, until obtaining a load of 1RM [10]. The bench press exercises were performed on the bench press of the FW–movement line, with the Olympic bar weighing between 16 and 18 kg, and the leg press exercises were performed on the 45° leg press of the bolt–movement line.

### 2.5. Anthropometric Evaluation 

To obtain the body mass, the TANITA^®^ digital scale was used, and the participant was instructed to remain standing in the center of the scale, wearing light clothes and no shoes. Height was measured using an inextensible tape measure fixed to a wall without a baseboard. The participant remained in an upright position looking forward and with arms extended alongside the body, barefoot, leaning the back of the neck, buttocks and heels on the wall where the measuring tape was. The body mass index was obtained through the ratio between the body mass in kilograms and the square of the height in meters and classified according to World Health Organization [11].

### 2.6. Food Intake Assessment 

For the collection of dietary data, the 24 h recall was performed, being applied once for each participant, by a trained nutritionist. The household measurements were converted into values of grams and milliliters according to the household measurements table [12], and, later, the data were analyzed and interpreted using the Dietbox^®^ version 2.0 Software, which presented as results the total energy, macro- and micronutrients. At the beginning of the study, participants were instructed not to change their dietary pattern during data collection.

### 2.7. Caffeine Intake Assessment 

To assess the caffeine intake by individuals, the Caffeine Consumption Questionnaire was applied, which aims to analyze the concentrations of caffeine in each product and/or foodstuff [13]. Through this questionnaire, information was collected on the weekly consumption of foods and products that are sources of caffeine, amount in grams or milliliters, consumption times for each of them and frequency of ingestion.

### 2.8. Statistical Analyses

The data were analyzed using the IBM SPSS software, version 20.0. The normality of the variables was evaluated by the Shapiro–Wilk W test, and the results are described as mean, median (minimum–maximum) and standard deviation of the mean. The differences between the means of the RM tests performed at the two evaluation points were obtained by Student’s t-test for dependent samples. The chi-square test was performed to assess the participants’ error and success when asked which supplement they were taking. A significance level of 5% (*p* < 0.05) was adopted, and the effect size was evaluated according to Cohen’s d test [14] (d < 0.2 “trivial”, 0.2 < d < 0.5 “small”, 0.5 < d < 0.8 “medium” and d > 0.8 “large”).

## 3. Results

Ten men practicing resistance exercise with a median training experience of 1.5 years and a mean body mass index of 24.4 ± 2.9 kg/m² were evaluated (Table 1). Regarding food intake, an average caloric intake of 2743.4 ± 828.0 kcal was observed, corresponding to 19.2% proteins, 46.9% carbohydrates and 33.0% lipids; and median caffeine consumption was 106.4 milligrams per day (Table 1).

In the analysis of life habits and environmental factors, three variables were considered: experience in years with resistance exercise, hours of sleep, for which an average of 6.7 ± 1.6 h per day was found, and mean temperature on the days of intervention, corresponding to 26.8 ± 6.4 °C (Table 1).

**Figure 2:**
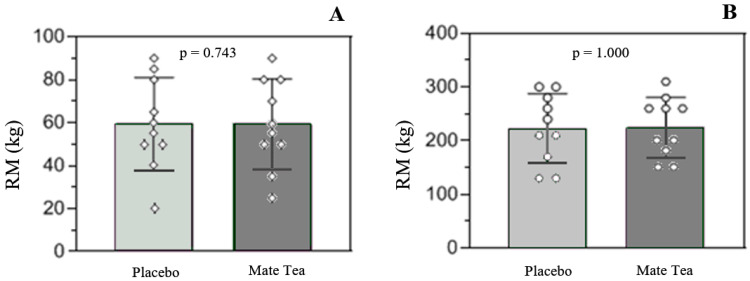
Load results (kg) in the 1RM protocols, comparing the interventions (placebo vs. mate tea). (**A**) RM result for the bench press exercise. (**B**) RM result for the leg press exercise. Values presented as mean and standard deviation.

There was no difference when comparing the type of intervention in either the 1RM leg press test (placebo: 223 ± 64.3 kg vs. YM: 225 ± 56.2 kg, *p* = 0.743) or in the test of the 1RM bench press (placebo: 59.5 ± 21.5 kg vs. YM: 59.5 ± 20.7 kg, *p* = 1.000), with a trivial effect size (Cohen’s d = 0.03 and d = 0, respectively) (Figure 2).

At the end of the study, participants were asked what they believed they were taking. On the first day of evaluation, 10% believed they had taken a placebo, 50% YM, and 40% did not know how to respond. On the second day of evaluation, 20% believed they had taken a placebo, 70% YM, and 10% did not know how to respond, showing no difference between right and wrong (*p* = 0.14). In addition, no side effects were reported by the participants during the intervention period.

## 4. Discussion

We observed that the YM supplementation compared to the placebo did not influence muscle strength following the strength muscle tests using the bench press and leg press. However, unlike in our work, in which we offered 3 g of YM one hour before the tests, Panza et al. [8], who offered fractional supplementation of 3 g of YM (1 gram, three times a day), found a positive outcome in the recovery of muscle strength 24 h after a session of eccentric exercises in non-athletes. These different outcomes suggest that the effect of YM fluctuates depending on the frequency of tea consumption or the type of exercise performed, having a greater influence on the process of muscle strength recovery.

The method of culture and preparation of YM can change the concentration of bioactive compounds in the drink, such as xanthine caffeine [15], which is responsible for the pre-workout ergogenic effect [16]. Likewise, studying and evaluating the degree of purity and roasting of mate tea is important to minimize the loss of bioactive compounds. Another aspect that should be evaluated is whether prolonged consumption of YM leads to ergogenic resistance, since this result has been observed when isolated caffeine (3 mg/kg/day) is used for more than four weeks [17,18]. 

During our study, participants stopped consuming foods rich in caffeine in order to avoid any interference. However, Gonçalves et. al. [19], evaluating the performance of cyclists after supplementation of (6 mg/kg/day) for seven days, found that the effect of acute caffeine supplementation on performance was not influenced by the habitual consumption of caffeine in the diet. This result suggests that there is no influence of dietary caffeine consumption on YM supplementation.

We noticed in our work that the average number of hours of sleep of the participants was 6.7 ± 1.6 h per day, which is below the recommendations of seven or more hours per day [20]. The problem with the reduced number of hours of sleep is due to the increase in IL-6 and TNF-α levels, which corroborate a pro-inflammatory state, which can interfere in sports performance [21]. In athletes with a history of sleep deprivation, a reduction in reaction time, reduced ability to understand, retention and implementation of training strategies are often observed, in addition to an increase in errors in situations of pressure or urgency [21]. Corroborating these findings, a systematic review concluded that sleep extension promotes beneficial effects in some aspects in the performance and recovery of athletes [22]. However, Knowles et al. [23] raised the concept that sleep deprivation during resistance training had little effect on muscle strength, with greater impairment on strength in multi-joint movements.

Within this perspective, the history of few hours of sleep reported by the participants of our study may have contributed to a decline in performance when applying the RM protocols, leading to lighter loads. However, as in this study the general average of hours of sleep was collected, regardless of the day of each intervention, we do not know the impact of sleep on each moment of the intervention. However, as there was no difference in test loads between the placebo and YM, we suggest that the impact of sleep deprivation affected the entire intervention period of the study. Thus, further studies are recommended to follow the individuals’ sleep history for each intervention day to assess this outcome.

Regarding the ambient temperature on the intervention days, whose average was lower than 30 °C, the participants reported a feeling of heat during the strength tests, because the place where the tests were carried out was cooled with fans. The sensation of heat during physical practice can increase fatigue [24] and compromise resistance exercise [25]. Considering the temperatures reached in the environment of this study, it was estimated that the sensation of heat may have led to the onset of muscle fatigue, resulting in lower loads borne in the exercises, which reinforces the importance of controlled environments in order to minimize bias. The practicalities in conducting this research and the possibility of intervention in a short period of time were positive points, which contributed to the fact that there were no dropouts during the study. In addition, the strengths of this work are the standardization of the pre-test snack and the usual assessment of food intake. As there is no recommendation for the daily consumption of YM, and knowing that very high doses become unpalatable, which can cause gastric irritation, anxiety, insomnia and tachycardia [26], we used the same dose as in the study by Panza et al. [8], where no side effects were stated.

We also emphasized that conducting the research during the COVID-19 pandemic made it difficult to recruit participants, which justifies the small sample size and the variation in the experience of the participants with resistance training, which may have been a bias in this work. As limitations, we point out that we did not perform biochemical evaluation of inflammatory markers and we cannot guarantee that the caffeine and bioactive compounds from YM were absorbed prior to the muscle tests. 

In conclusion, YM acute consumption before strength muscle tests likely does not influence the load lifted by physically active men. Further studies should be carried out, with a larger sample size and controlling for bias, in order to quantify the serum level of inflammatory markers, YM and active compounds, as well as to analyze the effect of different concentrations of caffeine in mate tea on muscle strength and identify the ergogenic action time of YM supplementation following the resistance exercise.

## Figures and Tables

**Table 1:** Sample characterization and variables of interest.

Anthropometric Measurements	
Weight (kg)	70.2 ± 9.8
Height (m)	1.7 ± 0.07
Body mass index (kg/m²)	24.4 ± 2.9
**Food Intake**	
Total kilocalories (kcal)	2743.4 ± 828.0
Total protein (g/day	132.0 ± 48.8
Protein/kg/dia (g/day)	2.0 ± 0.5
Total carbohydrates (g/day)	321.6 ± 118.3
Total lipids (g/day)	100.7 ± 43.9
Caffeine (mg) *	106.4 (0–467.5)
**Life Habits and Environmental Factor**	
Sleep (hours)	6.7 ± 1.6
Training experience (years)	1.5 (0.2–8)
Temperature (°C) (days of intervention)	26.8 ± 6.4

Values are presented as mean and standard deviation; * Values presented as median (minimum–maximum).

## Supplementary Materials

The following are available online at https://www.mdpi.com/article/10.3390/nu14132619/s1, Figure S1: Intervention period. RM: maximum repetition.

## References

[1] Valenzuela P.L., Morales J.S., Emanuele E., Pareja-Galeano H., Lucia A. (2019). Supplements with purported effects on muscle mass and strength. Eur. J. Nutr..

[2] Bracesco N., Sanchez A., Contreras V., Menini T., Gugliucci A. (2011). Recent advances on Ilex paraguariensis research: Minireview. J. Ethnopharmacol..

[3] Da Veiga D., Bringhenti R., Copes R., Tatsch E., Moresco R., Comim F., Premaor M. (2018). Protective effect of yerba mate intake on the cardiovascular system: A post hoc analysis study in postmenopausal women. Braz. J. Med Biol. Res..

[4] Marrelli M., Conforti F., Araniti F., Statti G.A. (2016). Effects of Saponins on Lipid Metabolism: A Review of Potential Health Benefits in the Treatment of Obesity. Molecules.

[5] Gambero A., Ribeiro M.L. (2015). The Positive Effects of Yerba Maté (Ilex paraguariensis) in Obesity. Nutrients.

[6] Alkhatib A., Atcheson R. (2017). Yerba Maté (Ilex paraguariensis) Metabolic, Satiety, and Mood State Effects at Rest and during Prolonged Exercise. Nutrients.

[7] Sellami M., Slimeni O., Pokrywka A., Kuvačić G., Hayes L.D., Milic M., Padulo J. (2018). Herbal medicine for sports: A review. J. Int. Soc. Sports Nutr..

[8] Panza V.P., Diefenthaeler F., Tamborindeguy A.C., Camargo C., De Moura B.M., Brunetta H.S., Sakugawa R.L., De Oliveira M.V., Puel E.D.O., Nunes E.A. (2016). Effects of mate tea consumption on muscle strength and oxidative stress markers after eccentric exercise. Br. J. Nutr..

[9] Cases J., Romain C., Marín-Pagán C., Chung L.H., Rubio-Pérez J.M., Laurent C., Gaillet S., Prost-Camus E., Prost M., Alcaraz P.E. (2017). Supplementation with a Polyphenol-Rich Extract, PerfLoad®, Improves Physical Performance during High-Intensity Exercise: A Randomized, Double Blind, Crossover Trial. Nutrients.

[10] Sakamoto A., Sinclair P.J. (2006). Effect of Movement Velocity on the Relationship Between Training Load and the Number of Repetitions of Bench Press. J. Strength Cond. Res..

[11] World Health Organization (1998). Physical Status: The Use and Interpretation of Anthropometry.

[12] Pinheiro A.B.V., Lacerda E.M.A., Benzecry E.H., Gomes M.C.S., Costa V.M. (2009). Tabela para Avaliaçao de Consumo Alimentar em Medidas Caseiras.

[13] Irons J.G., Bassett D.T., Prendergast C.O., Landrum R.E., Heinz A.J. (2016). Development and Initial Validation of the Caffeine Consumption Questionnaire-Revised. J. Caffeine Res..

[14] Cohen J. (1988). Statistical Power Analysis for the Behavioral Sciences.

[15] Duarte M.M., Tomasi J.D.C., Helm C.V., Amano E., Lazzarotto M., De Godoy R.C.B., Nogueira A.C., Wendling I. (2020). Caffeinated and decaffeinated mate tea: Effect of toasting on bioactive compounds and consumer acceptance. Rev. Bras. Ciencias. Agrar..

[16] Vitale K., Getzin A. (2019). Nutrition and Supplement Update for the Endurance Athlete: Review and Recommendations. Nutrients.

[17] Lara B., Ruiz C., Salinero J.J., Del Coso J. (2019). Time course of tolerance to the performance benefits of caffeine. PLoS ONE.

[18] Beaumont R., Cordery P., Funnell M., Mears S., James L.J., Watson P. (2016). Chronic ingestion of a low dose of caffeine induces tolerance to the performance benefits of caffeine. J. Sports Sci..

[19] Gonçalves L.S., Painelli V.S., Yamaguchi G., De Oliveira L.F., Saunders B., Da Silva R.P., Maciel E., Artioli G.G., Roschel H., Gualano B. (2017). Dispelling the myth that habitual caffeine consumption influences the performance response to acute caffeine supplementation. J. Appl. Physiol..

[20] Watson N.F., Badr M.S., Belenky G., Bliwise D.L., Buxton O., Buysse D., Dinges D.F., Gangwisch J., Grandner M.A., Kushida C. (2015). Recommended Amount of Sleep for a Healthy Adult: A Joint Consensus Statement of the American Academy of Sleep Medicine and Sleep Research Society. Sleep.

[21] Fullagar H.H.K., Skorski S., Duffield R., Hammes D., Coutts A.J., Meyer T. (2014). Sleep and Athletic Performance: The Effects of Sleep Loss on Exercise Performance, and Physiological and Cognitive Responses to Exercise. Sports Med..

[22] Bonnar D., Bartel K., Kakoschke N., Lang C. (2018). Sleep Interventions Designed to Improve Athletic Performance and Recovery: A Systematic Review of Current Approaches. Sports Med..

[23] Knowles O.E., Drinkwater E., Urwin C.S., Lamon S., Aisbett B. (2018). Inadequate sleep and muscle strength: Implications for resistance training. J. Sci. Med. Sport.

[24] Flood T.R. (2018). Menthol Use for Performance in Hot Environments. Curr. Sports Med. Rep..

[25] Racinais S., Alonso J.M., Coutts A.J., Flouris A.D., Girard O., González-Alonso J., Hausswirth C., Jay O., Lee J.K.W., Mitchell N. (2015). Consensus recommendations on training and competing in the heat. Br. J. Sports Med..

[26] Klein G., Stefanuto A., Boaventura B.C.B., De Morais E.C., Cavalcante L.D.S., De Andrade F., Wazlawik E., Di Pietro P.F., Maraschin M., Da Silva E.L. (2011). Mate tea (Ilex paraguariensis) improves glycemic and lipid profiles of type 2 diabetes and pre-diabetes individuals: A pilot study. J. Am. Coll. Nutr..

