# Supplementary figures and images for: Acute Supplementation of Yerba Mate Extract Did Not Change Muscle Strength in Physically Active Men Following the Strength Muscle Test: A Pilot Clinical Trial
Source: Nutrients. 2022 Jun 24;14(13):2619. doi: 10.3390/nu14132619 (PMC9268497; doi:10.3390/nu14132619)

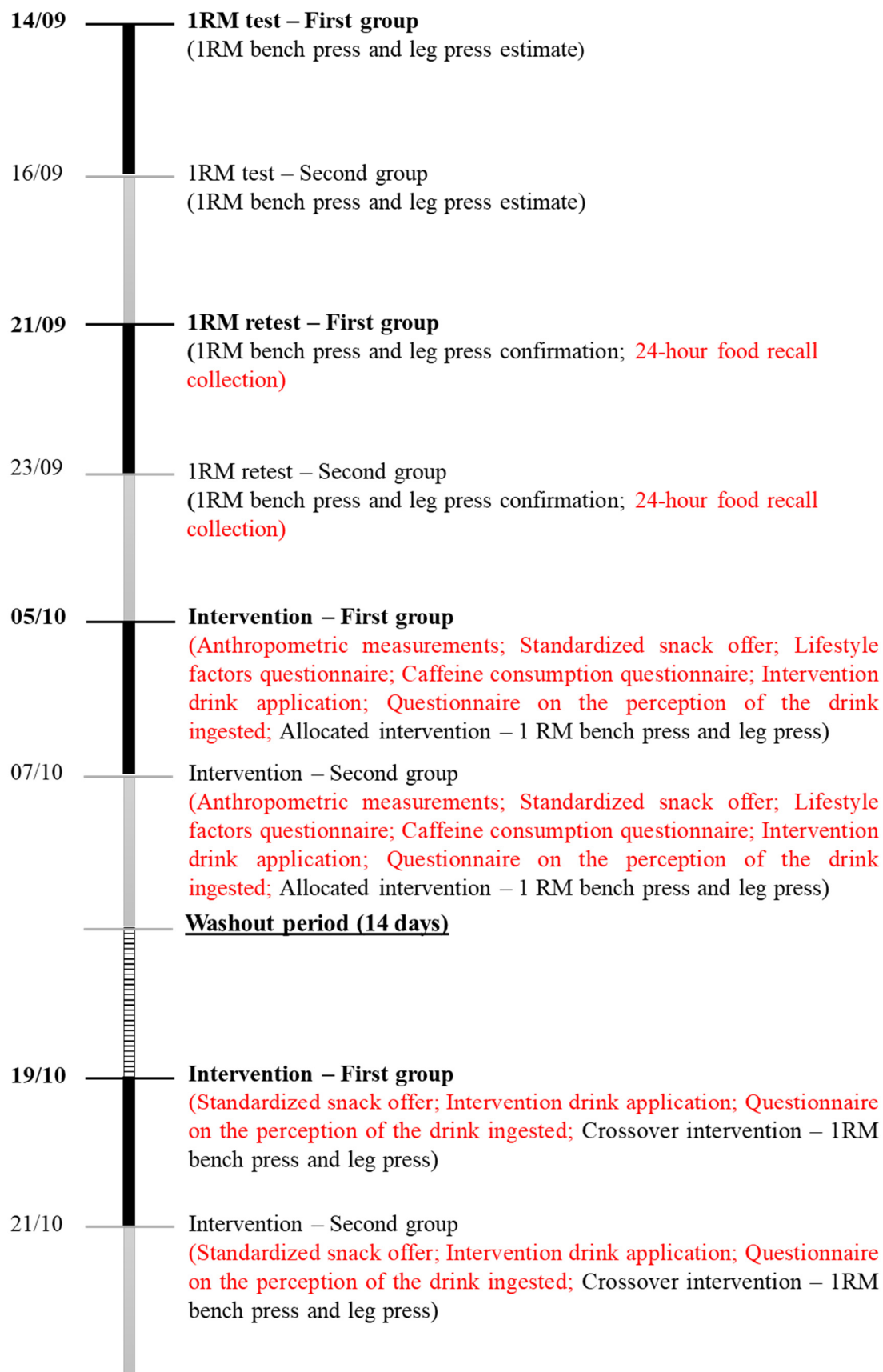

**Supplementary Figure S1.** Intervention period. RM: maximum repetition

Supplement: Supplementary file 1 [file nutrients-14-02619-s001.zip › nutrients-1703734-supplementary.pdf]
